# Relationship between Children’s Intergenerational Emotional Support and Subjective Well-Being among Middle-Aged and Elderly People in China: The Mediation Role of the Sense of Social Fairness

**DOI:** 10.3390/ijerph19010389

**Published:** 2021-12-30

**Authors:** Yixuan Liu, Liumeng Li, Guomei Miao, Xinyan Yang, Yinghui Wu, Yanling Xu, Yonghong Gao, Yongzhi Zhan, Yiwei Zhong, Shujuan Yang

**Affiliations:** Department of Social Medicine and Health Management, School of Public Health, Jilin University, Changchun 130021, China; liuyx20@mails.jlu.edu.cn (Y.L.); lilm19@mails.jlu.edu.cn (L.L.); Miaogm20@mails.jlu.edu.cn (G.M.); xinyan21@mails.jlu.edu.cn (X.Y.); wuyh21@mails.jlu.edu.cn (Y.W.); ylxu21@mails.jlu.edu.cn (Y.X.); Gaoyh19@mails.jlu.edu.cn (Y.G.); zhanyz20@mails.jlu.edu.cn (Y.Z.); zhongyw21@mails.jlu.edu.cn (Y.Z.)

**Keywords:** subjective well-being (SWB), the sense of social fairness, children’s intergenerational emotional support, middle-aged and elderly people, CGSS

## Abstract

This study explored the relations between children’s intergenerational emotional support and subjective well-being (SWB) among Chinese middle-aged (45–60 years old) and elderly people (over 60 years old) and the mediation effect of the sense of social fairness between such associations. Using the 2017 Chinese General Social Survey dataset, a nationally representative survey, we selected 2413 middle-aged and elderly people who are 45 years and older, who suited the study requirements with 1097 males and 1316 females, which accounts for 45.5% and 54.5%, respectively. The average of age was 61.62 years old (SD = 10.45). The mediation analyses were conducted by multivariate regression and the SPSS macro PROCESS program. The results indicated that there was a significant association between children’s intergenerational emotional support and subjective well-being of middle-aged and elderly people (β = 0.0819, *p* < 0.001). Children’s intergenerational emotional support also had an indirect impact on subjective well-being through one path: the mediating role of the sense of social fairness (0.012). Direct effect (0.0699) and mediation effect (0.012) accounting for the total effect proportion was 85.35% and 14.65%, respectively. The findings may offer some meaningful implications for improving subjective well-being of middle-aged and elderly people. Future research should pay attention to the aforementioned factors with more detailed and comprehensive studies.

## 1. Introduction

Population aging is a global trend that likely occurs throughout the 21st century [1]. Most countries in the world, including China, are facing the severe challenge of population aging [2]. As the second largest global economy, China currently houses the world’s largest population of 1.4 billion (19.13% of the world population) and is rapidly transforming into an aging nation [3]. China’s population aged 60 and over accounted for 18.70 percent, up 5.44 percent from the sixth national census in 2010, according to data from the seventh national census [4]. The quality of life, mental health and other problems of middle-aged and elderly people gradually has become a hot topic of high concern in the whole society under the background of population aging.

Subjective well-being (SWB) refers to an individual’s overall and comprehensive assessment of life quality according to their own standards, which is subjective, stable and holistic [5]. SWB could be measured by the assessment of how frequently or intensely people experience a variety of positive and negative emotions, such as “happiness,” “sadness,” “anger,” or “joyfulness” [6]. Subjective well-being involves individuals’ subjective appraisals of their life from their own perspective [7,8]. According to Diener and Suh, “SWB research is concerned with individuals’ subjective experiences of their lives. The underlying assumption is that well-being can be defined by people’s conscious experiences—in terms of hedonic feelings or cognitive satisfactions. The field is built on the presumption that to understand the individuals’ experiential quality of well-being, it is appropriate to directly examine how a person feels about life in the context of his or her own standards” [9]. As an important comprehensive indicator to measure people’s quality of life and mental health, SWB is a major concept of interest for augmenting other economic and social measures of welfare [10] and has attracted more and more attention [11]. It also provides people with a meaningful and complementary measure of health, when compared to objective measures. Improving the wellbeing of middle-aged and elderly people is emerging as a key societal aspiration, which is related to family harmony, social stability and national development [12]. After achieving basic life securities, subjective well-being of most middle-aged and elderly people comes from individual health status, family and interpersonal relationship and subjective feelings about individual surroundings. As the special group that is about to enter old age and has already entered old age, middle and older age are the times when well-being is particularly important [13]. High level of subjective well-being can not only promote the long-term development of physical and mental health and self-perfection of middle-aged and elderly people, but also be considered as an important indicator of successful aging, which contributes to the realization of healthy aging [14]. Therefore, it is of great significance to pay attention to the SWB of middle-aged and elderly people [13,14,15,16].

Intergenerational support is an informal form of intergenerational exchange, which includes economic, life care and emotional support between offspring and parents [17]. In essence, it is the resource flow and exchange behavior between parents and children based on consanguinity and kinship. Intergenerational support from family continues to be a primary source of care and support for old people in many developing countries motivating sustained attention to the relationship between social support and well-being of elderly people [18]. Filial piety is an important manifestation of Confucianism that obliges children to respect, care for, and revere their parents [19,20]. In China, an individual’s perception of family relationships is mainly influenced by the filial piety, a central concept in Confucian culture [21]. Meanwhile, other eastern countries, such as Japan, also have strong norms of family obligation and filial norms about caring for older parents [22]. Thus, family members, especially adult children, are expected to take care of and support the elderly, and the family support seems to become an important source of living satisfaction and well-being for aging adults [23,24]. Some studies have reported positive correlations between intergenerational or familial support and subjective well-being [25] and mental health among older adults [26,27,28], whereas others have reported that high levels of support from adult children is either harmful [29] or has a negligible effect on the well-being of older adults [30]. A positive intergenerational relationship, such as frequent intergenerational exchanges, can alleviate loneliness and depression symptoms and promote the elderly’s mental health [31,32,33]. Merz and Consedine suggested that emotional support is generally associated with higher well-being, whereas instrumental support is related to decreased well-being [24]. A study by Lai et al. [34] reported that a stronger sense of closeness with grandchildren was associated with self-rated health status and quality of life among older Chinese immigrants in the United States. Meaningful communication between elderly people and their adult children likely benefit the mental health of the former [35]. A German study by Mahne and Huxold [36] reported that contact frequency and emotional closeness with grandchildren boosted positive aspects of subjective well-being. On the basis of a sample of elderly women in Taiwan, China, Lin et al. suggested that stronger emotional bonds with adult children increased older women’s life satisfaction [37]. In rural China, elderly people receiving economic support from their children can improve their negative emotions and reduce depression [38]. Compared with material and economic intergenerational support, non-economic intergenerational support, such as emotional communication and consolation between middle-aged and elderly people and their children, is more conducive to enhancing their happiness, and can positively improve the subjective well-being of middle-aged and elderly people.

The sense of social fairness is a kind of subjective feeling derived from psychology and emotion. People often compare their expectations and self-actualization with others to get their own subjective cognition and judgment of perceived fairness [39,40,41]. As Lin pointed out, social judgment is emotional, and judgment often has a strong emotional association [42]. Individuals’ perception of social fairness is an important part of their social cognition and judgment, which is inevitably affected by emotional factors. Although there are few studies on the influence of emotional support on the sense of social fairness, some studies have shown that emotional expressions can have strong effects on cognition [43,44]. Spapé et al. [45] investigated how emotional expressions modulate the difference between processing of fair and unfair offers, which is also referred to as fairness perception [46]. For Chinese, the household environment and traditional cultures still affect Chinese people [47]. Children have an irreplaceable position in the eyes of the middle-aged and elderly people, especially in terms of spiritual solace and emotional experiences. As one aspect of emotional support, children’s emotional intergenerational support for middle-aged and elderly parents to some extent reflects parents’ emotional expression. We think that it can also influence parents’ perception of fairness to a certain extent. Therefore, in addition to the direct impact of children’s intergenerational emotional support on subjective well-being of the middle-aged and elderly people, the research on the potential mechanism in this path is also very meaningful. Studies on different populations show that higher individual’s sense of fairness is beneficial to improving an individual’s mental health [48]. From an equity theory perspective, several researchers have emphasized the importance of reciprocal support [49,50]. At the same time, fairness heuristic theory also holds that the sense of fairness is formed by heuristics, and when individuals make fair judgments, their sense of fairness is associated with their psychological state [51], which is closely related to subjective well-being. Many empirical studies have found that residents’ subjective well-being would be significantly enhanced with social fairness [52,53,54]. Considering the association of the sense of social fairness with children’s intergenerational emotional support, and subjective well-being of the middle-aged and elderly people, we hypothesized that children’s intergenerational emotional support might have an impact on the sense of social fairness and that the sense of social fairness might be a potential mediator between children’s intergenerational emotional support and subjective well-being among middle-aged and elderly people.

A large number of studies have proved that subjective well-being of middle-aged and elderly people is affected by family and individual factors such as age, gender, religious faith, education, marital status, economic status, and self-rated health [55,56,57,58,59]. Some cross-sectional and longitudinal studies have indicated that subjective well-being presents a U-shaped state in the whole life cycle and increases in older age (around age 40–50 years old) [60,61]. Moreover, this pattern of a U-shaped association with a dip in midlife is seen across many countries around the world [62]. Evidence suggests that being married or living with a partner can have a positive effect on life satisfaction and is associated with a higher well-being [63,64]. Pinquart et al. found that socioeconomic status is positively correlated with the subjective well-being of people [65]. Health and subjective well-being were also found to be positively and significantly related [66]. For middle-aged and older people, close and supportive relationships are extremely important in reducing negative effects and rebuilding well-being [67], which are related to individual family size [68]. In view of this, it is necessary for our study to take into consideration some sociodemographic characteristics control variables including age, gender, religious faith, education level, and marital status, which may have an association with subjective well-being of middle-aged and elderly people.

Combing the literature on factors affecting the subjective well-being of middle-aged and elderly people, it can be seen that previous studies in China focus on the influence analysis of factors such as individual characteristics and social structure on subjective well-being. In the existing studies on “intergenerational support of children” and “subjective well-being of parents”, most of them reported that there was a simple linear correlation between them separately or intuitively, lacking detailed discussions on some mediation variables [69,70]. Whether children’s intergenerational emotional support will directly or indirectly affect the subjective well-being of middle-aged and elderly people in China remains unknown, which further highlights the significance to investigate an association between them. The research objects of this study are middle-aged and elderly people. According to the new classification of age by the World Health Organization, the young age is from 25 to 44, middle age is 44–60, elderly age is 60–75, senile age is 75–90 and long-livers are after 90 [71]. Referring to relevant literature, we found that most of the studies define people aged 45 and above as middle-aged and elderly population [72,73,74]. Based on this, we took middle-aged people (45–60 years old) and elderly people (including “elderly age (60–75 years old)”, “senile age (75–90 years old)” and “long-livers (over 90 years old)” as the research object to explore the influence of children’s intergenerational emotional support on their middle-aged and elderly parents’ subjective well-being. Meanwhile, from the perspective of social fairness, we analyzed the internal correlation among children’s intergenerational emotional support, the middle-aged and elderly people’s sense of social fairness and subjective well-being. We will also test mediation effect of the sense of social fairness between children’s intergenerational emotional support and subjective well-being in Chinese middle-aged and elderly population. Therefore, to verify these questions, we made the following hypotheses. Firstly, there would be a relationship between children’s intergenerational emotional support and subjective well-being of middle-aged and elderly people in China. Secondly, the sense of social fairness would have a mediation effect between children’s intergenerational emotional support and subjective well-being.

## 2. Materials and Methods

### 2.1. Data Source and Sample

The data in this study came from the Chinese General Social Survey (CGSS) in 2017. CGSS is the earliest national, comprehensive, and continuous academic survey project in China. It adopts multistage stratified sampling, which is currently recognized as representative data with scientific research value in academia [75]. The research objects are middle-aged people (45–60 years old) and elderly people (over 60 years old). After screening and eliminating samples with incomplete variables, 2413 valid respondents were yielded in our study.

### 2.2. Measurements

#### 2.2.1. Independent Variable

In this study, intergenerational emotional support is shown by the frequency of the spiritual comfort provided by children to their middle-aged and elderly parents. In the CGSS questionnaire, the main predictor is, “In the past year, how often have your adult children who are closest to you listened to your worry or thoughts?”, and the self-rated was recorded on a five-point scale as follows: 1 = never, 2 = rarely, 3 = sometimes, 4 = often, and 5 = very frequently.

#### 2.2.2. Dependent Variable

Subjective well-being was measured by the five-point Likert-type scale for the question: “In general, do you think your life is happy?” with responses including: “1 (very unhappy)”, “2 (unhappy)”, “3 (general)”, “4 (happy)”, “5 (very happy)”.

#### 2.2.3. Mediating Variable

In this study, we took people’s subjective perception of social fairness as an important indicator to reflect the sense of social fairness among middle-aged and elderly people. The main predictor is, “In general, do you think today’s society is fair?” Respondents’ answers ranged from 1 (very unfair), 2 (relatively unfair), 3 (average), 4 (relatively fair), and 5 (very fair).

#### 2.2.4. Control Variables

The survey collected demographic information including region (the central and western regions = 0, east region = 1), gender (female = 0,male = 1), age, registered residence (rural = 0, city and town = 1), religious faith (no = 0, yes = 1), education level (elementary school and below = 1, junior high school = 2, high school or technical secondary school = 3, junior college = 4 bachelor or above = 5), marital status was categorized as married and not married (single/cohabitation/divorced/widow), (not married = 0, married = 1), family economic status (well below average = 1, below average = 2, average = 3, above average = 4, well above average = 5), self-rated health (very bad = 1, bad = 2, general = 3, good = 4, very good = 5), endowment and medical insurance (no = 0, yes = 1), and household size and the number of children.

### 2.3. Data Analysis

In this study, IBM SPSS Statistics version 24 was used to complete all the data analysis and processing. We conducted a descriptive analysis to describe the basic sociodemographic characteristics of the study population, and a correlation analysis to verify the relationship between variables. In order to test whether there was mediation effect of the sense of social fairness between children’s intergenerational emotional support and subjective well-being of their middle-aged and elderly parents, we used multivariate regression analyses and the SPSS macro PROCESS program (Model 4) [76]. A *p*-value of 0.05 was considered statistically significant. The study set bootstrap confidence interval (CI) at 95% based on 5000 bootstrapped samples. If zero was not included in the interval of 95% CI, it indicated that the mediation effect was significant.

## 3. Results

### 3.1. Characteristics of Samples

Table 1 shows the demographic characteristics of the participants. In our study, middle-aged people aged 45–60 accounted for 48.1% of the subjects, and the elderly people aged 61 and above accounted for 51.9%. The average of age was 61.62 years old (SD = 10.45). In terms of regional distribution, middle-aged and elderly people from central and western regions and eastern regions accounted for 58.2% and 41.8%, respectively. In general, the survey samples were evenly distributed in terms of age, region, gender ratio and household type. In terms of marital status, as the respondents were middle-aged and elderly, the proportion of married people was high, accounting for 79.7%, while those who were not married account for 20.3%. In terms of health, most people consider themselves to be in relatively good health. In terms of family economic situation, the overall level was below the middle level, and most people were not quite satisfied with their family economic situation. As far as social security is concerned, the vast majority of middle-aged and elderly people have participated in social endowment insurance and medical insurance. Different demographic variables were taken as influencing factors of subjective well-being of middle-aged and elderly people to conduct Chi-square test, and the results were shown in Table 1. Chi-square test revealed significant differences in subjective well-being of middle-aged and elderly people across the age, region, registered residence, education level, marital status, family economic status, self-rated health, endowment insurance, medical insurance and the number of sons.

### 3.2. Preliminary Analyses

The correlations between examined variables are presented in Table 2. The results of the correlation analysis were consistent with our expected hypotheses, and all the analysis results were statistically significant at the level of *p* < 0.05 (two-tailed). First, the intergenerational emotional support of children was positively correlated with subjective well-being and sense of social fairness of middle-aged and elderly people significantly, with correlation coefficients of 0.098 and 0.048, respectively. Secondly, there was a significant positive correlation between subjective well-being of the middle-aged and elderly people and their sense of social fairness (r = 0.312).

### 3.3. Testing for Mediation Effect

**Figure 1:**
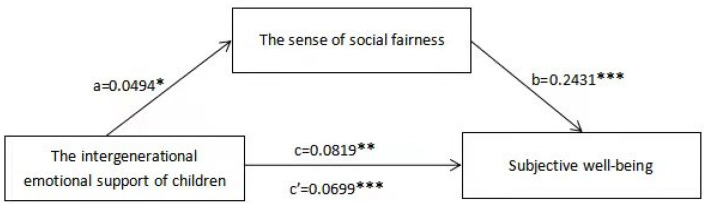
The conceptional framework of the mediation model. Note: * *p* < 0.05, ** *p* < 0.01, *** *p* < 0.001.

The mediation analysis result of the sense of social fairness on the intergenerational emotional support of children and subjective well-being of middle-aged and elderly people are shown in Figure 1, Table 3 and Table 4. First, three independent regression models were conducted to examine the mediation effects of the sense of social fairness on the associations between children’s intergenerational emotional support and subjective well-being of middle-aged and elderly people. As shown in Table 3, Model 1 reported that the total effects of the intergenerational emotional support of children on subjective well-being was significant (β = 0.0819, *p* < 0.001) after controlling for all the demographic variables, which meant the higher the frequency of the emotional connection provided by children to their parents, the happier their middle-aged and elderly parents felt. Model 2 showed that children’s intergenerational emotional support had a significant association on social fairness (β = 0.0494, *p* < 0.05). Model 3 indicated the relationship among the children’s intergenerational emotional support, the sense of social fairness and subjective well-being. The results indicated that social fairness had a significant positive effect on subjective well-being (β = 0.2431, *p* < 0.001). The intergenerational support of children still had a significant positive correlation with subjective well-being after adding the sense of social fairness (β = 0.0699, *p* < 0.001), but the size decreased compared to its original association in Model 1. In addition, as shown in Figure 1 and Table 4, the SPSS macro PROCESS program showed that the upper and lower bounds of bootstrap 95%confidence interval of the direct effect of children’s intergenerational emotional support to subjective well-being and the mediating effect of perceived social fairness didn’t include 0, which also indicated that children’s intergenerational emotional support could not only directly affect subjective well-being, but also affect subjective well-being through the mediating effect of perceived social fairness. Direct effect (0.0699) and mediation effect (0.012) accounted for the total effect proportion was 85.35% and 14.65%, respectively. See Figure 1, Table 3 and Table 4 for more information. 

## 4. Discussion

In this study, the main goal was to examine whether the sense of social fairness could mediate the relationship between the intergenerational emotional support of children and subjective well-being among Chinese middle-aged (45–60 years old) and elderly people (over 60 years old). Firstly, the results showed that the intergenerational emotional support of children significantly affected the subjective well-being among middle-aged and elderly people. Second, the sense of social fairness played a mediating effect between the intergenerational emotional support of children and subjective well-being of Chinese middle-aged and elderly people. Therefore, our results confirmed the hypotheses which we made in the beginning of the study.

### 4.1. The Direct Effect of the Intergenerational Emotional Support of Children on Subjective Well-Being

The results suggested that the intergenerational emotional support of children was positively correlated with subjective well-being of Chinese middle-aged and elderly people. The conclusion of this research was similar to the results of Li [77], which reported that the mutual emotional support between children and older people in rural China was associated with improved subjective health of older women. The study from China by Jia and He also found that intergenerational emotional support has a positive effect on the health of the elderly living alone and living at home, respectively [78]. Subjective well-being of middle-aged and elderly people increased significantly with the emotional support of their children. As the elderly grow older, their physical function and emotional state will change greatly, and their social role is also changing to a certain extent. At the same time, while people are getting older and increasingly perceive time as finite, they place more emphases on emotional meaning in their life rather than those goals that expand their horizons but to be achieved with emotional burden [79]. Emotional intimacy among individuals promotes support and helping one another [80]. The emotional exchange between children and parents can reflect the emotional state and degree of intimacy between the elderly and children. Children often listen to their parents’ thoughts, which can help release middle-aged and elderly people’s negative emotional pressure, promote their physical and mental health and experience, meet their emotional needs and expectations to a certain extent, and thus improve their subjective well-being. Therefore, children should pay attention to the health of their middle-aged and elderly parents, including mental and physical health. They also should give enough emotional security, care and love to their parents through emotional communication to help them eliminate negative emotions, enhance their own value identity in social interaction and life, which can improve the middle-aged and elderly people’s sense of social fairness and subjective well-being.

### 4.2. The Mediation Effect of the Sense of Social Fairness

In the relationship between children’s intergenerational emotional support and subjective well-being of middle-aged and elderly people, the sense of social fairness played a mediating role partially. The sense of social fairness is often accompanied by some emotional experiences [81]. As a way for parents to express their personal feelings, emotional support provided by children can reduce the impact of negative emotions on the mental health of middle-aged and elderly people and produce positive emotional experiences, which affect individuals’ cognition and judgment of events. The more emotional support parents receive from their children, the more secure they feel from their families. This gives people a good sense of control over their surroundings and encourages them to have confidence in the future. Therefore, children’s emotional intergenerational support brings psychological satisfaction and positive emotional experiences to middle-aged and elderly parents, which to some extent promotes their sense of social fairness. High emotional intimacy and strong social support are conducive to the health and well-being of older adults [82]. Based on the economic principles of cost/benefit, social exchange theory is often used to explain the relationship between intergenerational support and life satisfaction of adults in later life, suggesting that people will have higher levels of well-being when support received is greater than support provided due to the maximized benefits and minimized losses in relationships with others [83,84]. The increase of emotional communication between children and their parents makes middle-aged and elderly parents receive tremendous spiritual comfort and support, enhance their confidence about self-acceptance and social activities. It encourages middle-aged and elderly people to integrate into social interaction actively and makes the subjective feelings about their social fairness better. In general, children’s emotional intergenerational support greatly satisfies the emotional needs of the middle-aged and elderly people, and thus improves their sense of social fairness, which enriches their psychological resources and raises their subjective well-being greatly in a way. Using these studies, children need to take effective measures to coordinate the relationship with their parents. We should change social prejudice for middle-aged and elderly people, maintain the relative fairness of society, enhance their sense of social belonging and enthusiasm for participating in social activities. Furthermore, the relevant government needs to increase investment in basic public services, optimize social resources, build a more comprehensive social fairness system, improve public services in medical care, education and employment, and raise middle-aged and elderly people’s subjective well-being by improving people’s livelihood.

## 5. Conclusions

This study provides evidence of the influence of children’s intergenerational emotional support on subjective well-being among middle-aged and elderly people aged 45 and above in China, and it also suggests that the mediation effect of the sense of social fairness between such associations occurred. The findings of the present study shed light on the significance of children’s intergenerational emotional support and the sense of social fairness in the subjective well-being of Chinese middle-aged and elderly people. The results of this study also enrich our theoretical and practical understanding about the influence of children’s intergenerational emotional support and the sense of social fairness on the subjective well-being among middle-aged and elderly people. We call for further research evidence from more detailed and comprehensive studies with larger samples to verify their deeper causality and prove our findings.

## Figures and Tables

**Table 1:** Demographic characteristics of the participants (N = 2413).

Variables	Category	N	%	χ^2^	*p*
Age	45–60	1160	48.1	20.477	<0.001
	61and above	1253	51.9		
Gender	Female	1316	54.5	3.211	0.523
	Male	1097	45.5		
Region	The central and western regions	1405	58.2	39.525	<0.001
	East region	1008	41.8		
Registered residence	Rural	1291	53.5	58.272	<0.001
	City and town	1122	46.5		
Religious faith	No	2120	87.9	3.992	0.407
	Yes	293	12.1		
Education level	Elementary school and below	1119	46.4	36.988	<0.01
	Junior high school	713	29.5		
	High school	380	15.7		
	Junior college	125	5.2		
	Bachelor or above	76	3.1		
Marital status	No	489	20.3	15.177	<0.01
	Yes	1924	79.7		
Family economic status	Well below average	175	7.3	27.240	<0.05
	Below average	789	32.7		
	Average	1261	52.3		
	Above average	178	7.4		
	Well above average	10	0.4		
Self-rated health	Very bad	152	6.3	291.447	<0.001
	Bad	526	21.8		
	General	734	30.4		
	Good	754	31.2		
	Very good	247	10.2		
Endowment insurance	No	498	20.6	21.132	<0.001
	Yes	1915	79.4		
Medical insurance	No	163	6.8	13.929	<0.01
	Yes	2250	93.2		
Family size				44.692	0.281
sons				53.519	<0.001
Daughters				40.872	0.265

**Table 2:** Descriptive statistics and correlation among variables.

Variables	The Intergenerational Emotional Support of Children	Subjective Well-Being	The Sense of Social Fairness
The intergenerational emotional support of children	1		
Subjective well-being	0.098 **	1	
The sense of social fairness	0.048 *	0.312 **	1

Note: * *p* < 0.05, ** *p* < 0.01.

**Table 3:** Mediating model testing of the sense of social fairness.

Variables	Subjective Well-Being (Model 1)		The Sense of Social Fairness (Model 2)		Subjective Well-Being (Model 3)	
	β	t	β	t	β	t
Age	0.2410	6.3470 ***	0.2357	4.8878 ***	0.1837	5.0602 ***
Gender	−0.0751	−2.1793 *	0.0043	0.0987	−0.0762	−2.3228 *
Region	0.0549	1.4405	−0.1610	−3.3286 ***	0.0940	2.5885 **
Registered residence	0.1231	3.1719 **	−0.0522	−1.0579	0.1358	3.6766 ***
Religious faith	0.0125	0.2372	0.0321	0.4797	0.0047	0.0936
Marital status	0.1189	2.6649 **	−0.1236	−2.1828 *	0.1489	3.5061 ***
Family economic status	0.0041	0.1822	0.0164	0.5707	0.0001	0.0063
Self-rated health	0.2362	14.4475 ***	0.0938	4.5203 ***	0.2134	13.6614 ***
Endowment insurance	0.0583	1.3061	0.0711	1.2539	0.0410	0.9657
Medical insurance	0.1810	2.5440 *	0.1387	1.5352	0.1472	2.1748 *
Family size	0.0202	1.6028	−0.0073	−0.4553	0.0220	1.8325
Sons	0.0257	1.1757	0.0656	2.3636 *	0.0097	0.4682
Daughters	0.0277	1.5468	0.0515	2.2665 *	0.0152	0.8894
The intergenerational emotional support of children	0.0819	4.8333 ***	0.0494	2.2948 *	0.0699	4.3310 ***
The sense of social fairness					0.2431	15.8931 ***
R^2^	0.129		0.041		0.212	
F	25.365 ***		7.336 ***		42.997 ***	

Note: * *p* < 0.05, ** *p* < 0.01, *** *p* < 0.001.

**Table 4:** Table of total effect, direct effect and mediation effect.

Pathway	Effect	BootSE	BootLLCI	BootULCI
Total effect	0.0819	0.0169	0.0487	0.1151
Direct effect	0.0699	0.0161	0.0383	0.1016
Indirect effects	0.0120	0.0055	0.0015	0.0231

## Data Availability

Publicly available datasets were analyzed in this study. This data can be found here: http://cnsda.ruc.edu.cn/index.php?r=projects/view&id=94525591 (accessed on 15 August 2021).
